# Achieving Chemical Accuracy in Cyclodextrin Host–Guest Binding via Integrative Atomistic Modelling

**DOI:** 10.1002/advs.202519782

**Published:** 2025-12-14

**Authors:** Xiaohui Wang, Linqiong Qiu, Hongyu Wang, Wenting Tang, Jiayang Leng, John Z. H. Zhang, Piero Procacci, Zhaoxi Sun

**Affiliations:** ^1^ Faculty of Synthetic Biology Shenzhen University of Advanced Technology Shenzhen 518107 China; ^2^ Faculty of Biosciences Taizhou Technician College Zhejiang 318000 China; ^3^ Beijing Key Laboratory of Digital Media School of Computer Science and Engineering Beihang University Beijing 100191 China; ^4^ Department of Biomedical Engineering Southern University of Science and Technology Shenzhen Guangdong 518055 China; ^5^ Shanghai Frontiers Science Center of Artificial Intelligence and Deep Learning and NYU‐ECNU Center for Computational Chemistry NYU‐Shanghai 1555 Century Avenue Pudong New Area Shanghai 200062 China; ^6^ Department of Chemistry New York University NewYork NY 10003 USA; ^7^ Dipartimento di Chimica “Ugo Schiff” Università degli Studi di Firenze Via della Lastruccia 3 Sesto Fiorentino 50019 Italy

**Keywords:** binding Mode, cyclodextrin, force field development, host‐guest chemistry, nonequilibrium fast switching

## Abstract

Cyclodextrins are amphiphilic macromolecular containers that are particularly valuable in applications ranging from biomedicine to environmental science. Despite years of development of computational techniques for cyclodextrin host‐guest coordination, accurate modelling of these supramolecular systems remains challenging. In this work, an integrative computational technique is presented to solve this problem. The protocol integrates a force‐field recalibration procedure, equilibrium enhanced sampling, nonequilibrium Hamiltonian switching, a work convolution algorithm, a series of finite‐size corrections, and energy decomposition analysis. A large‐scale survey of 222 CD host–guest systems is performed to demonstrate that the protocol enables the fast calculation of cyclodextrin host‐guest binding strength without compromise in accuracy, an unbiased capture of cyclodextrin dynamics in the free CD, and the multi‐modal behavior of the coordination patterns, and further the identification of the physicochemical driving force stabilizing host‐guest complexes. Especially, the protocol consistently provides accurate results for various systems while conventional transferable force fields systematically fail for larger and more flexible 𝛾‐CD. It thus opens new opportunities for high‐throughput screening and rational design across diverse macrocyclic host families.

## Graphical abstract

A generalizable computational framework is presented that overcomes long‐standing challenges in modelling cyclodextrin host‐guest binding by integrating host‐specific force‐field refinement, equilibrium enhanced sampling, nonequilibrium alchemical switching, convolution sampling of independent works, rigorous finite‐size corrections, and QM‐based interaction analysis. The approach achieves chemical accuracy, exposes the limitations of transferable force fields, and enables predictive design of cyclodextrin‐based delivery systems and functional materials.

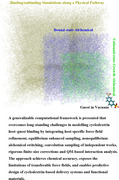

## 1 Introduction

Cyclodextrins (CDs) are a prominent class of macromolecular containers that have attracted enormous attention owing to their capability for tunable encapsulation of therapeutic agents and controllable release.^[^ ^1^, ^2^, ^3^, ^4^ ^]^ Among various macrocyclic receptors, such as calixarenes, cucurbit[*n*]urils, and pillar[*n*]arenes, CDs stand out as the most widely employed members, thanks to their ready availability, low cost, and favorable solubility profiles. Structurally, CDs are cyclic oligosaccharides composed of 𝛼‐(1,4)‐linked glucose subunits, which create a hydrophobic internal cavity surrounded by a hydrophilic exterior. This dual characteristic enables CDs to act as versatile carriers and reservoirs in pharmaceutical, food, cosmetic, and environmental applications.^[^ ^5^, ^6^ ^]^ The most bio‐safe and naturally abundant CDs are 𝛼‐CD (6 glucose units), 𝛽‐CD (7 units), and 𝛽‐CD (8 units), which have different cavity sizes and their host–guest selectivity. E.g., 𝛾‐CD can encapsulate larger bioactive molecules and is particularly valuable in pharmaceutical and nutraceutical applications.

Despite the years of development of many computational techniques, accurate modeling of CD host‐guest systems remains a challenge in modern computational chemistry. For example, some recent benchmarks on end‐point free energy calculations in their naïve and QM‐treatment‐involved forms report an accuracy level >2 kcal mol^−1^.^[^ ^7^, ^8^ ^]^ Strategies incorporating higher‐level statistical mechanics treatments (e.g., alchemical free energy techniques) reduced the errors, but still did not achieve the chemical‐accuracy level.^[^ ^9^, ^10^ ^]^ Aside from the binding affinity, the reliable determination of the host‐guest encapsulation pattern (i.e., the complex structure) remains also a key challenge. An incorrect binding mode would not only mislead the understanding of CD host‐guest complexations, but also introduce systematic errors of unknown magnitude to the computed binding affinity. A sound pose prediction in host‐guest complexes relies on both an accurate description of the energetics of the host and/or guest molecule(s) and a powerful sampling strategy to explore the high‐dimensional space of the intra‐ and intermolecular packing. At present, there are obstacles in both aspects. First, conventional transferable force fields are inherently limited in their ability to describe host–guest systems, as their parameterization is generally derived from systems that differ fundamentally from macrocyclic hosts.^[^ ^11^, ^12^, ^13^ ^]^ Second, the critical role of the conformational flexibility of both host and guest molecules has also been observed in many host‐guest complexes, e.g., the multi‐modal binding behaviors in cucurbiturils and pillararenes,^[^ ^14^, ^15^ ^]^ which brings great difficulties for configurational sampling. Note that specialized regimes like machine learning predictors for binding affinities^[^ ^16^, ^17^ ^]^ are out of the scope of the current work, as these models cannot provide the binding affinity and the binding mode, i.e., the whole picture of CD host‐guest binding simultaneously.

Accurate modelling of CD host‐guest binding requires the rigorous or high‐quality treatment of all aspects of computational modelling. First, the Hamiltonian used to describe the host‐guest system needs to be accurate. Unfortunately, as we would show in the lateral section of this manuscript, this is not really achievable with mainstream general‐purpose force fields predominantly applied in modern computational studies. Second, a thorough exploration of the conformational space should be performed to secure a reliable description of the intra‐ and intermolecular packing pattern, i.e., the binding mode. The unbiased sampling strategy widely applied in end‐point free energy calculations (e.g., MM/PBSA) is obviously unable to achieve a good sampling quality, and it seems necessary to incorporate some type of enhanced sampling techniques in CD host‐guest modelling. Third, given an accurate description of the energetics inside the system and a reliable complex structure, we also need a rigorous algorithm to compute the binding strength of the host‐guest pair. In our previous works, we tried to solve these problems by introducing a protocol incorporating a refitted force field as Hamiltonian and a spherical‐coordinates‐biased metadynamics method for conformational sampling. However, the method suffers from costly sampling of the binding/unbinding event in a large simulation cell, posing difficulties in both data storage and computational burden. In this manuscript, we present the central result as an efficient integrative computational strategy resolving all these problems by combining force‐field recalibration, equilibrium enhanced sampling, nonequilibrium alchemical fast‐switching transformation, the work convolution treatment for more efficient data usage, a batch of finite‐size corrections to recover the targeted thermodynamic ensemble, and a QM‐based energy decomposition analysis for the nature of host‐guest interaction. Ultimately, this integrative computational modelling protocol leads to a chemical‐accuracy probe for CD host‐guest binding.

## 2 Result and Discussion

### 2.1 An Integrative Modelling Protocol for CD Host‐Guest Modelling

**Figure 1:**
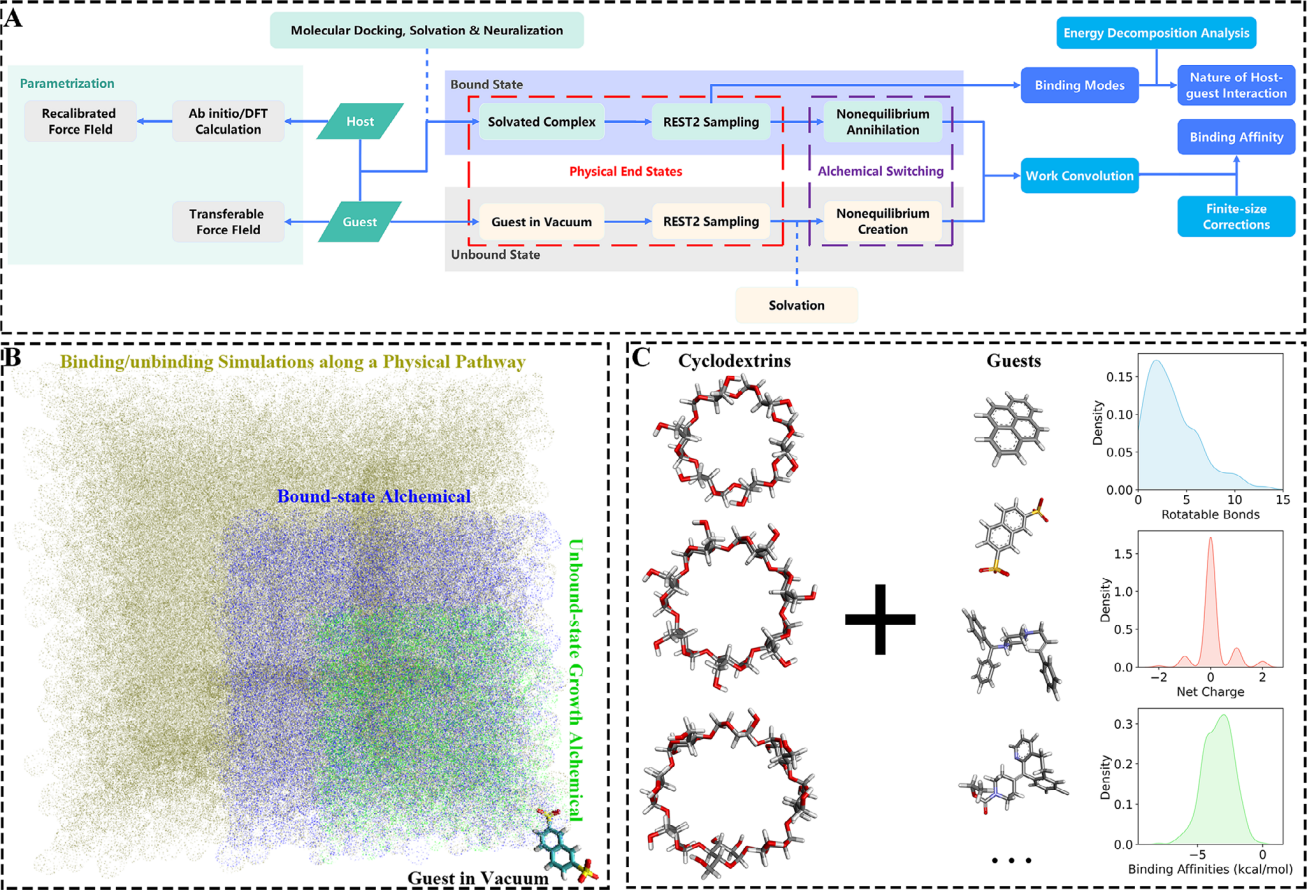
A) The integrative workflow for accurate modelling of cyclodextrin host‐guest binding. B) Comparison between simulation cells involved in our integrative workflow (green, blue, and black) and those when directly simulating the binding/unbinding event along a physical pathway (brown). C) CD host‐guest binding considered in the current work involves three prototypical 𝛼‐, 𝛽‐, 𝛾‐CDs and a batch of drug‐like guests (a total of 74, as listed in Figures S1–S3, Supporting Information), along with the physicochemical properties of the guest molecules (binding affinities, number of rotatable bonds, and net charges).

The proposed integrative modelling strategy for CD host‐guest binding incorporates several key components shown in **Figure** **1**A, including the development of high‐accuracy force fields for the macrocyclic hosts, the bound‐unbound leg separation with each involving equilibrium end‐state enhanced sampling and nonequilibrium alchemical annihilation/creation, post‐simulation free energy extraction and corrections, and energy decomposition analysis for the nature of host‐guest interaction. We provide a brief description to key components here, and more details are given in Section 4.

As transferable force fields were developed for drug‐like molecules, they provide a good description of the targeted molecules (guests). However, they have never seen huge macrocycles and the corresponding unique chemical environments, which consequently leads to their problematic descriptions of these species. We therefore refit transferable force fields for these macrocyclic hosts based on DFT energetic and force data, and secure a high‐accuracy description of these species.

With a recalibrated set of force‐field parameters, the absolute binding affinity calculation is then divided into two stages, i.e., the bound and unbound legs. In each stage, instead of wasting computational time in the non‐physical alchemical states, we distributed most sampling in the end states to effectively explore the binding‐mode space. This is achieved with the equilibrium enhanced sampling method REST2,^[^ ^18^ ^]^ with which a batch of equilibrium host‐guest and guest‐only configurations representative of individual equilibrium ensembles is secured. Then, a swarm of nonequilibrium fast‐switching simulations is initiated to accumulate the microscopic nonequilibrium work. The independent works in the two stages/legs are combined in a procedure named work convolution ^[^ ^19^, ^20^ ^],^ the post‐simulation finite‐site correction is added, and the final equilibrium free energy difference (the binding affinity) is extracted. At this stage, both the thermodynamic and structural perspectives about the host‐guest complexation are secured, which is further augmented through a QM‐based energy decomposition analyses that explore the nature of the host‐guest interaction. Such an integrative modelling protocol enables a thorough characterization of the host‐guest interaction in the solvated phase, which provides an effective tool for high‐throughput screening, mechanism elucidation, and rational design.

### 2.2 Higher Efficiency Compared with Modern Protocols

Compared with our tailored approach for host–guest interactions, existing protocols achieving similar goals differ mostly in free energy calculations and force‐field descriptions (Hamiltonians). Concerning the former, the popular protocols that directly explore the binding/unbinding events along a physical pathway^[^ ^14^, ^15^, ^21^ ^]^ require the simulation of the large brown cell shown in Figure 1B, since the definition of the unbound state in the physical space requires a clear host‐guest separation (a large host‐guest distance). By contrast, the current regime divides the sampling into three smaller boxes (blue and green regions for solvated bound and unbound phases and the stick representation with black text for the gas‐phase guest‐only simulation). Consequently, the current alchemical‐based scheme achieves a noticeable acceleration in typical cases (see Table S1, Supporting Information). A note to add is that, unlike the enhanced sampling strategies directly sampling the binding/unbinding events in the configurational space, the current alchemical‐based protocol cannot yield kinetic parameters (e.g., k_on_ and k_off_). Concerning the latter force‐field accuracy, based on a large‐scale survey of 222 diverse host‐guest systems (see Figure 1C) involving the three prototypical 𝛼‐, 𝛽‐ and 𝛾‐CD forms and 74 drug‐like guests (see Figures S1–S3 and Section S1, Supporting Information for details), we demonstrate the failure of modern transferable practices and emphasize the necessity of securing customized high‐accuracy parameter sets in host‐guest modelling.

### 2.3 True Dynamics of the Macrocyclic CD

**Figure 2:**
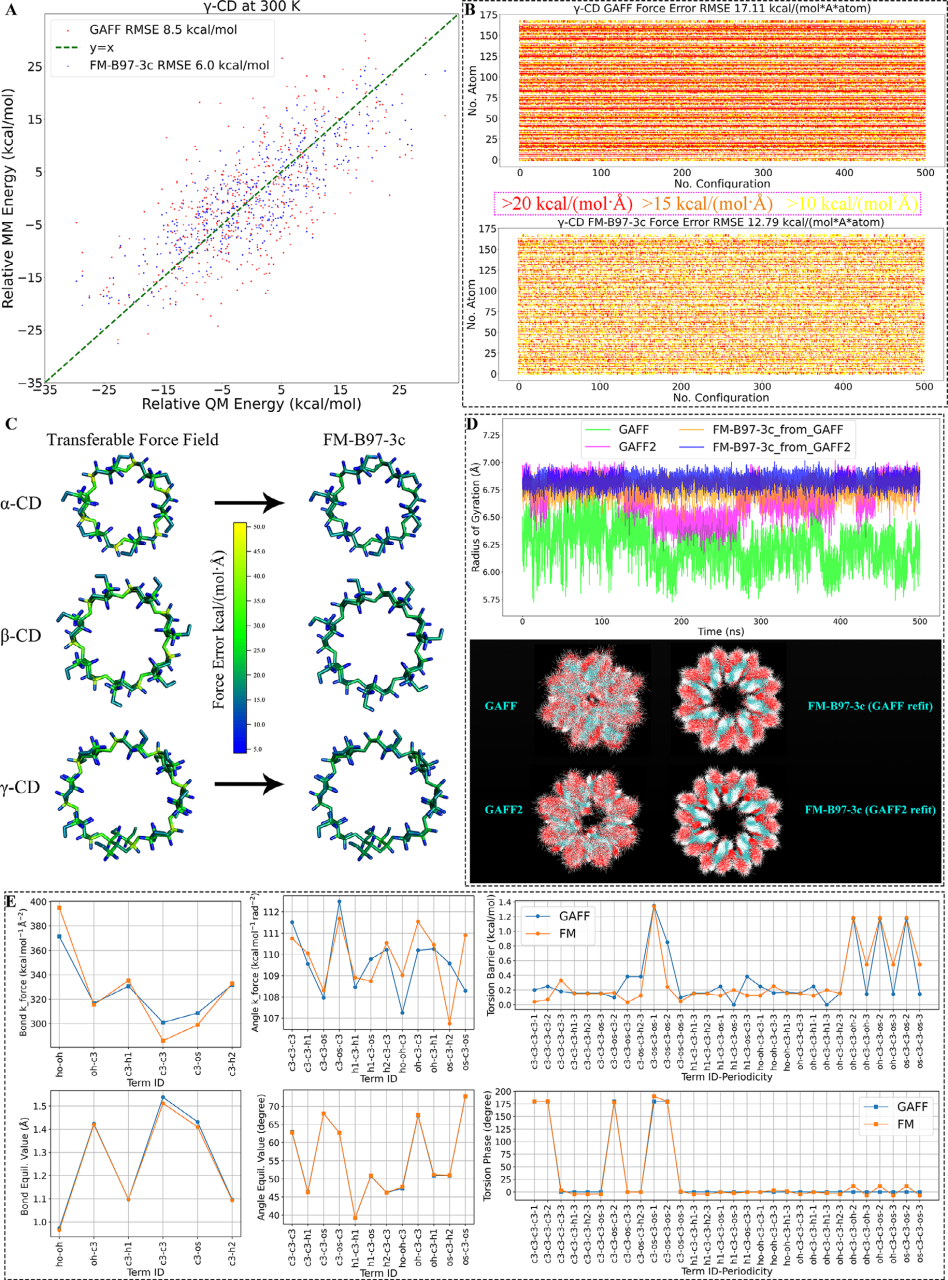
A) The errors/deviations of the energetics for 𝛾‐CD produced by transferable GAFF and refitted parameter sets. B) The time series of the force errors (Frobenius norm) for 𝛾‐CD in the 300 K evaluation sampling. C) By‐atom mapping of the force errors (‖Δ ***F**_i_* ‖_2_) for 𝛼‐, 𝛽‐, and 𝛾‐CD. D) The radius of gyration of 𝛾‐CD under different parameter sets and the structure overlap during the 500 ns sampling. E) Term‐specific comparison between transferable GAFF (blue) and refitted molecule‐specific FM (orange) parameters for cyclodextrins. The suffix periodicity is added to the identifier of torsional potentials, as there are multiple terms with different periodicity imposed on the same torsion.

While transferable force fields were predominantly applied in most CD host‐guest modelling, here we demonstrate that they are inaccurate in describing the behaviors of macrocycles, especially the prototype 𝛾‐CD. For each CD molecule, we fit high‐accuracy molecule‐specific parameters using the generalized force‐matching (FM) protocol.^[^ ^22^, ^23^ ^]^ We present the energy and force accuracies of the refitted FM‐B97‐3c and transferable GAFF parameters in **Figure** **2**A,B, where the superiority or the close‐to‐QM (the B97‐3c reference) behavior is obvious. Similar observations for all CD molecules are further confirmed by the by‐atom maps of force errors in Figure 2C, where GAFF introduces widespread inaccuracies across the macrocycle backbone, whereas the force field refitting sharply reduces these discrepancies.

Based on all parameter sets, we conducted a 500 ns sampling of the unbound host in water. The time series of the radius of gyration (Rg) and the structural overlay presented in Figure 2D clearly illustrate the consequences of these differences: transferable GAFF derivatives consistently yield overly flexible, softened, and most critically, asymmetric rings that collapse over time, effectively destroying the open cavity essential for guest recognition. By contrast, the refitted FM‐B97‐3c parameter sets, regardless of the initial guess used in refitting, maintain a stable, fully opened, and symmetric CD cavity throughout the trajectory. Such behavior under FM‐B97‐3c is in excellent agreement with recent experimental ^1^H NMR studies of CD dynamics,^[^ ^24^ ^]^ which report symmetric conformations in the solvated phase. The ability of FM to reproduce both QM reference energetics and experimental host dynamics strongly underscores its reliability in capturing the true dynamics of CDs, while the failures of transferable GAFF derivatives highlight the pitfalls of applying general‐purpose parameters to complex macrocyclic systems.

To rationalize the origin of these differences, we present the term‐specific comparison of GAFF and FM parameters in Figure 2E. Here, significant deviations in bond force constants, angle force constants, and torsional barriers emerge as the dominant sources of error in GAFF, ultimately leading to distorted host geometries and unstable binding cavities. In contrast, FM produces parameters that more faithfully reflect the underlying electronic structure, ensuring both structural integrity and dynamic stability across the CD family.

### 2.4 Higher Accuracy Compared with Modern Practices

**Figure 3:**
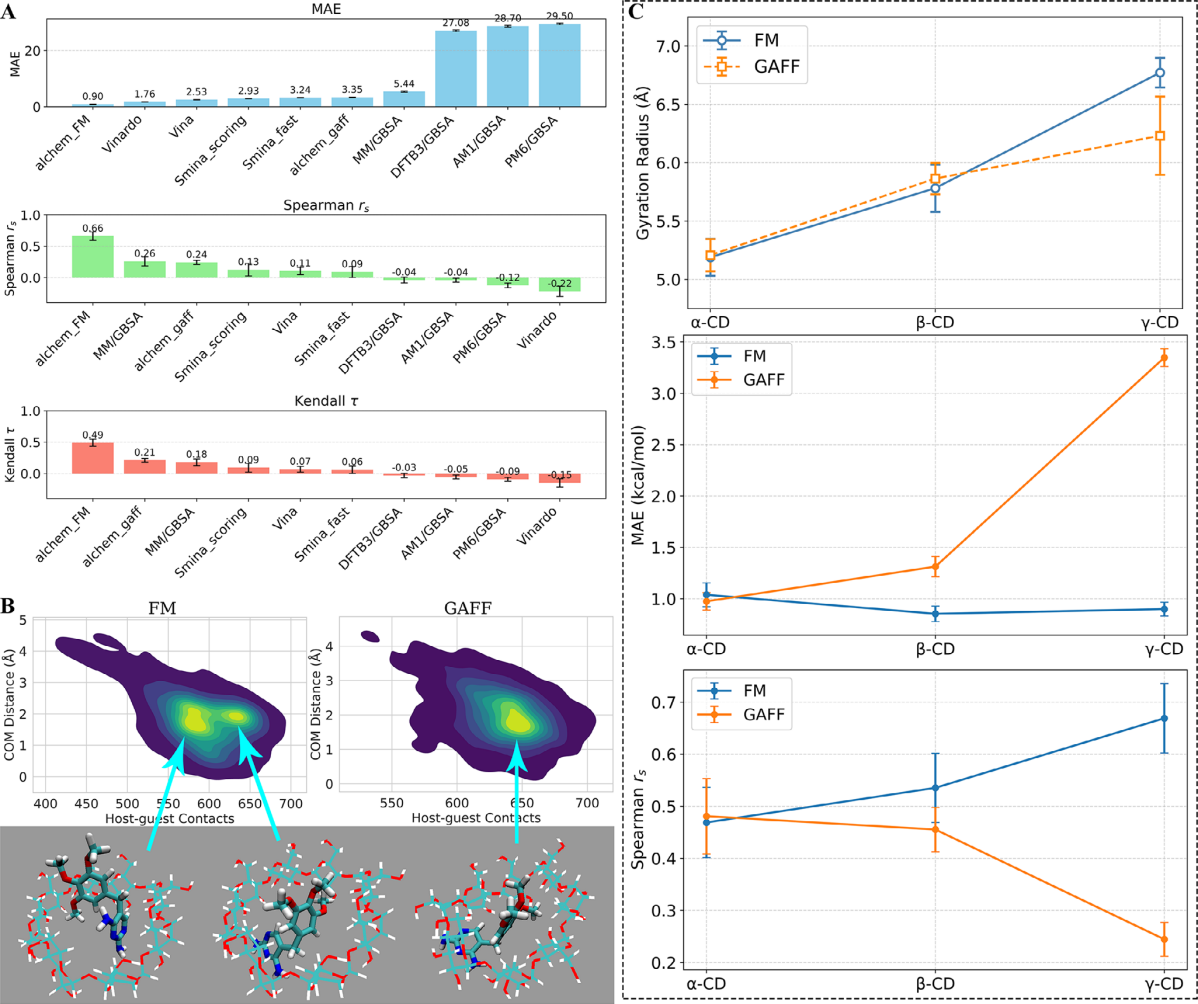
A) The performance on affinity prediction for 𝛾‐CD. Our alchemical‐involved integrative modelling protocol and various baselines (end‐point free energy calculations based on MM and semi‐empirical QM Hamiltonians and molecular docking protocols) were considered. Results for the 𝛼‐, 𝛽‐CD datasets are given in Figures S4 and S5 (Supporting Information). B) The free energy landscape and representative host‐guest encapsulation patterns for 𝛾‐CD explored in the equilibrium REST2 sampling under the refitted FM and transferable GAFF parameter sets. C) The gyration radius of the CD hosts and performance metrics of our calculated binding affinities for CD hosts with different numbers of repeating units.

In **Figure** **3**A and Figures S4 and S5 (Supporting Information), we present the performance metrics of our integrative modelling protocol (alchem_FM) along with many baseline schemes. Specifically, the alchem_gaff regime differs from our protocol in the force‐field parameters used to describe the macrocyclic host (the transferable GAFF is used here instead of our refitted FM parameter set). The MM/GBSA, AM1/GBSA, PM6/GBSA, and DFTB3/GBSA are robust end‐point free energy techniques widely employed in modern host‐guest modelling. The Vina and Vinardo are two protocols using the AutoDock Vina software with its default Vina and alternative Vinardo scoring functions, and smina_scoring and smina_fast are the two scoring regimes in the smina software. For all three host‐guest datasets, our alchem_FM protocol achieves a chemical‐accuracy prediction quality (MAE≈1 kcal mol^−1^) and the top ranking performance (Spearman and Kendall correlation coefficients), which suggests the robustness and predictive power of our integrative modelling protocol in host‐guest binding. Interestingly, for the smallest ring considered in this work, 𝛼‐CD, MM/GBSA achieves a ranking power comparable to our scheme, but it fails significantly for the reproduction of absolute values of binding affinities (i.e., according to its huge MAE).

### 2.5 Binding Mode and the Nature of Intermolecular Interaction

The force‐field parameters also have a significant impact on the binding‐mode space. For an arbitrarily selected guest‐𝛾‐CD pair, the end‐state sampled configurations are projected on the distance between the centers of masses (COM) and the host‐guest contacts $C_{\mathrm{group}-\mathrm{gues}t}=\sum_{i\in \mathrm{group}}\sum_{j\in \mathrm{guest}}\frac{1}{1+{\left(\frac{r_{ij}}{r_{0}}\right)}^{6}}$ with *r*_0_ =  6 Å in Figure 3B, where the noticeable differences between the two‐minima FM and single‐state GAFF free energy landscapes confirm the essential role of the macrocycle force field in determining the host‐guest binding pattern. According to the structural visualization, it was recognized that the two conformations under FM differ in the guest group coordinated at the central cavity. Unlike the similar stabilities under FM, only the larger‐contact conformation is stable under the transferable GAFF parameter set. Further, the multimodal binding behavior in the current CD case is also observed in many other host‐guest systems, suggesting the complexity of host‐guest interaction patterns.^[^ ^14^, ^15^ ^]^ The bound structures of all host‐guest pairs probed under the FM parameter set are provided in the supporting information for interested users to explore the detailed binding modes themselves.

Based on these bound conformations, we further extract the nature of the interactions through QM‐based energy decomposition analysis in Figure S6 (Supporting Information), where the electrostatic, correlation/dispersion, and polarization interactions contribute to stabilization effects while the exchange‐repulsion and desolvation interactions lead to destabilization effects. In parallel with these fundamental physical components, the complex also features stabilizing interactions like hydrogen bonds, e.g., between the ‐NH_2_ group of the guest and the rim oxygen atoms of the host, which provides a complementary chemical‐level interpretation of the binding. These hydrogen bonds arise from the favorable alignment of electrostatic and polarization contributions and are further reinforced by local dispersion interactions, giving rise to a recognizable and chemically intuitive binding motif. Together, the physical (electrostatics and dispersion) and chemical (hydrogen‐bond) perspectives provide a multi‐layered understanding of the host–guest interaction landscape.

### 2.6 Ring‐Size Dependence of Transferable Force Fields

Through systematic statistical analysis, we identified an interesting dependence of the performance on the CD ring size in Figure 3C. The Rg, which reflects the openness of the central cavity, yielded comparable values with GAFF and FM parameterizations for the smaller (𝛼‐CD) and medium‐sized (𝛽‐CD) rings. The corresponding binding affinity predictions also show similar levels of accuracy, as indicated by both the scoring (MAE) and ranking (Spearman) metrics.

In contrast, significant deviations emerge for the larger 𝛾‐CD. The GAFF parameterization predicts a reduced Rg, corresponding to a collapsed and asymmetric cavity, whereas FM parameters yield an open and symmetric conformation consistent with experimental observations. This difference in the unbound state geometry propagates to the bound state, where distinct binding modes are stabilized under the two force fields. Specifically, the GAFF tendency to describe a compressed cavity leads to altered binding preferences and weaker agreement with experiment, while FM preserves a realistic open cavity and produces more reliable binding energetics. The experimental Rg from SAXS experiments is measured to be 7.1 Å,^[^ ^25^ ^]^ which also suggests an open cavity and agrees with the FM simulations. Collectively, these results demonstrate that while transferable force fields may perform satisfactorily for smaller CDs, their limitations become pronounced for larger ring systems, highlighting the critical role of accurate host flexibility in determining binding thermodynamics.

### 2.7 Broader Applicability and Future Directions

While the present study focuses on the prototypical 1:1 host‐guest binding mode, realistic cyclodextrin systems (particularly larger hosts such as 𝛾‐CD) often exhibit more complex behavior, including the accommodation of multiple guest molecules within a single cavity. The integrative strategy developed here is, in principle, fully capable of treating such non‐1:1 stoichiometries. In parallel, solvent identity represents another fundamental variable that shapes binding thermodynamics. Specifically, explicit‐solvent simulations within our workflow inherently capture competitive solvation, cavity hydration, and solvent‐mediated entropic effects, all of which can shift the balance between alternative binding modes. Although a comprehensive exploration of stoichiometry and solvent dependence lies beyond the scope of the current study, both aspects represent natural extensions of the present framework. Systematically designed benchmark datasets spanning a range of host‐guest dimensions and solvents will be essential for establishing statistically reliable trends, and these developments constitute important directions for future work.

## 3 Concluding Remarks

In this work, we introduced an integrative computational framework that achieves chemical‐accuracy characterization of CD host–guest binding by unifying force‐field recalibration, enhanced end‐state sampling, nonequilibrium alchemical transformations, efficient work convolution, and QM‐based interaction analysis. This protocol resolves two longstanding challenges in cyclodextrin modelling: the unsuitability of transferable force fields for macrocyclic hosts and the difficulty of capturing the full conformational and energetic landscape underlying host–guest interactions. By tailoring force‐field parameters, concentrating sampling on equilibrium states, and linking physical end states with nonequilibrium alchemical switching, our method improves efficiency compared to direct physical‐pathway simulations, captures the correct CD dynamics in both the unbound and bound states, and yields binding free energies in close agreement with experimental expectations.

The comparison with widely used transferable force fields highlights a critical point: while general‐purpose Hamiltonians may suffice for small and medium‐sized CD rings, they systematically fail for larger, more flexible rings such as 𝛾‐CD, where host dynamics and cavity openness are decisive for binding thermodynamics. Our protocol demonstrates that accurate host‐specific parametrization is indispensable for reproducing the true structural and energetic behavior of these systems. Moreover, by integrating rigorous free energy calculations with energy decomposition analysis, the workflow provides a detailed picture of the stabilizing and destabilizing forces shaping host‐guest recognition.

Looking forward, combining improved Hamiltonians, enhanced sampling schemes, and AI‐driven prediction models offers promising avenues to further refine the computational description of cyclodextrin complexes. Such advances will not only narrow the gap between simulation and experiment but also provide predictive guidelines for rational CD design, thereby accelerating the discovery and optimization of CD‐based drug delivery systems and functional materials. Overall, this study establishes a generalizable and computationally efficient strategy for cyclodextrin modelling, bridging the gap between thermodynamic prediction and mechanistic understanding while opening new opportunities for high‐throughput screening and rational design across diverse macrocyclic host families.

## 4 Experimental Section

### Construction of the CD Host‐Guest System

The structures of all molecules were presented under investigation in Figures S1–S3 (Supporting Information). The experimental 1:1 affinities of host‐guest binding were extracted from the literature. The protonation states of all guest molecules were determined according to the pH condition that the experimental affinities were measured (pH ≈7.4) and the microscopic pKa values of ionizable sites predicted by ChemAxon and MolGpKa^[^ ^26^ ^]^, due to the established performance of the two pKa predictors in previous literature.^[^ ^14^, ^15^ ^]^

Then, each molecule was parameterized with a general AMBER procedure. Namely, the fixed‐charge model was obtained by combining restrained electrostatic potential (RESP) charges and general AMBER force field (GAFF). The RESP charge generation involves B3LYP/6‐31G* optimization in vacuo, a HF/6‐31G* ESP scan, and a multi‐step regularized least‐squares fitting. The bonded and vdW terms were extracted from GAFF derivatives. As observed in the analyses of host dynamics, given the same set of atomic charges, the differences between bonded terms in GAFF derivatives lead to different cyclodextrin dynamic behaviors. It was therefore considered additional sets of molecular force fields by recalibrating the existing parameter sets based on QM calculations (align with GAFF parametrization). The relevant numerical details were discussed in Section 4.2.

As the host‐guest binding ratio under consideration was 1:1, the simulation cell of the complex system was constructed by adding a single host‐guest pair, spherical monovalent ions (sodium or chloride ions)^[^ ^27^ ^]^ for neutralization and TIP3P^[^ ^28^ ^]^ water molecules for solvation. The initial configuration of the bound structure was not of utmost importance here, as an additional phase of enhanced sampling for conformational exploration was incorporated in the lateral simulations. The host and guest molecules together were packed by picking the top‐1 docked pose from the AutoDock Vina protocol with the Vinardo scoring function.^[^ ^29^, ^30^ ^]^ For the solvated complex system, a preliminary ns‐length equilibration (energy minimization, NVT, and NPT equilibrations) was conducted to reach an equilibrated state, after which equilibrium enhanced sampling was initiated. With equilibrium configurations accumulated from equilibrium simulations, nonequilibrium fast‐switching simulations were then performed along an alchemical path, and the free energy differences between equilibrium states were computed. More detailed discussions about the sampling procedure will be presented in Section 4.3.

Aside from the solvated complex, the unbound state was also modelled. This involves the ligand/guest in a water box. For such a system, the simulation cell, following a similar workflow and conducting a short equilibration, was constructed.

With LINCS constraints ^[^ ^31^ ^],^ the time step of dynamics propagation was set to 1.5 fs. The temperature was regulated with velocity rescaling^[^ ^32^ ^]^ at 298 K, and the pressure at 1 atm was controlled with the Parrinello‐Rahman barostat.^[^ ^33^ ^]^ Long‐range electrostatics were treated with the smooth Particle‐mesh Ewald method. The simulations were run with GROMACS 2020.6 and PLUMED 2.7.4.^[^ ^34^ ^]^

### Recalibration of the Transferable Force Field

The molecular force field employed in our work follows the general AMBER form, i.e.,

(1) $$\begin{array}{cc} & U=\sum_{bonds}k_{r}{\left(r-r_{0}\right)}^{2}+\sum_{angles}k_{\theta }{\left(\theta -\theta_{0}\right)}^{2}+\sum_{dihedrals}V_{\varphi }\left[1+cos\left(n\varphi -\gamma \right)\right]\\ & \;+\sum_{i}\sum_{i<j}\left[\frac{q_{i}q_{j}}{r_{ij}}+\frac{A_{ij}}{r_{ij}^{12}}-\frac{B_{ij}}{r_{ij}^{6}}\right] \end{array}$$

where individual terms were self‐explanatory. Due to the transferable nature of the GAFF derivatives, their accuracy in specific systems would be relatively low compared with molecule‐specific force fields. Further, their training sets include merely drug‐like molecules that were significantly different from the macrocyclic hosts. Therefore, the practical applicability or suitability of transferable parameter sets would be low in macrocyclic host‐guest binding. As observed in the main article, the host dynamics produced by GAFF derivatives were totally incorrect. It was therefore explored the possibility of achieving a higher‐accuracy or closer‐to‐QM description based on the given functional form of force fields.

The bonded parameters that were critical in determining the intramolecular conformational preferences were adjusted with a generalized force‐matching FM ^[^ ^22^, ^23^ ^]^ regime. The loss function of this optimization problem was designed to include molecular energetics (relative energy), atomic forces (‖Δ ***F**_i_* ‖_2_) and a regularization term, i.e.,

(2) $$Loss_{FM}=Loss_{energy}+Loss_{force}+Loss_{regularization}$$

Shift and scaling transformations were applied to individual loss terms in order to improve the statistical behavior of the optimization procedure, and an L2 restraint was used for the regularization term. The objective function can therefore be rewritten as

(3) $$\begin{array}{cc} & Loss_{FM}=\sum_{i}^{N_{conf}}\omega_{i}\frac{{\left(E_{i}^{ref}-E_{i}^{MM}\right)}^{2}}{var\left(E^{ref}\right)}\\ & \;+\frac{1}{3N_{atoms}}\sum_{i}^{N_{conf}}\omega_{i}\sum_{j}^{N_{atoms}}\left[\Delta F_{ij}^{T}\langle F_{ij}^{ref}⊗F_{ij}^{ref}\rangle \Delta F_{ij}\right]\\ & \;+\sum_{m}^{N_{params}}\omega_{regularization}\frac{{\left(p_{m}-p_{m}^{orig}\right)}^{2}}{\gamma_{m}^{2}} \end{array}$$

where *N_conf_* denotes the number of configurations in the force‐field recalibration, *N_atoms_* was the number of atoms, ω was the weight of individual terms, *i* denotes the *i*th configuration, *j* represents the *j*th atom, *p* represents parameters, *m* was the *m*th parameter, γ was the width of the priori distribution (Gaussian for L2), the superscript *ref* denotes the QM data, *MM* denotes the force‐field data, *orig* was the original parameter set (i.e., GAFF derivatives).

The L2 term restrains the parameter space in the neighborhood of the initial guess to avoid overfitting. The weighting regime for energy, force, and regularization terms was set to 1:1:0.16, which was an empirical value achieving an intermediate level of regularizations. Among all bonded parameters, bond‐stretching, angle‐bending, and the barrier height and phase parameters of the torsional potentials were adjusted.

The configurations for QM calculations were obtained with an adaptive sampling regime using the refitted parameter set, which consists of 600 configurations per iteration, 10 ps per configuration, and 15 iterations in total. The sampling temperature was selected as 600 K, in order to achieve a good coverage of relevant conformational space, as the relative population of different states was not the focus in the training stage. In the evaluation stage, the room‐temperature condition (300 K) was used in order to assess the accuracy level in the room‐temperature‐accessible region of the configurational space. The QM reference data were accumulated from B97‐3c^[^ ^35^ ^]^ calculations using the ORCA5^[^ ^36^, ^37^ ^]^ package.

### Equilibrium and Nonequilibrium Sampling Strategies for Affinity Estimates

The free energy calculation generally follows the workflow shown in Figure 1A. The core of the statistical mechanics in the workflow was the combination of equilibrium enhanced sampling techniques and a nonequilibrium Hamiltonian switching process along an alchemical pathway for the estimation of binding strengths. The calculation of the central thermodynamic quantity, the binding affinity of host‐guest pairs Δ*G_bind_*, was decomposed into the calculations of guest‐environment interactions in the presence and absence of the host molecule (i.e., host‐guest encapsulation) Δ*G_calc_* and a couple of finite‐size corrections accounting for volume corrections Δ*G_volume_* and net‐charge variations Δ*G_charge_*,

(4) $$\Delta \;G_{bind}=\Delta G_{calc}+\Delta G_{volume}+\Delta G_{charge}$$

First, one or several reliable complex structures of the host‐guest pair need to be explored. To achieve this goal, using the AutoDock‐produced bound structure as a reasonable starting point, the REST2^[^ ^18^ ^]^ protocol was employed that includes the host and guest molecules in the hot region, an excessive list of replicas (32) to achieve a high exchange rate and thus short round‐trip times, and a high‐temperature limit of 1500 K. The acceptance ratio can thus be written as

(5) $$P_{acc}=min\left(1,e^{-\beta \left\{\left(\gamma_{j}-\gamma_{i}\right)\left[E_{hot}\left(x_{i}\right)+E_{cold}\left(x_{i}\right)\right]+\left(\gamma_{i}-\gamma_{j}\right)\left[E_{hot}\left(x_{j}\right)+E_{cold}\left(x_{j}\right)\right]\right\}}\right)$$

where *E* was the potential energy, β was the reciprocal temperature ($\frac{1}{k_{B}T}$ at 300 K), and $\gamma_{i}=\frac{\beta }{\beta_{i}}\;was$ the inverse temperature scaling factor that was chosen as a geometric series. This enables an unconstrained exploration of relevant conformational space, both for the intramolecular conformational preference (the macrocyclic host and the drug‐like guest) and for the intermolecular coordination patterns. The sampling length for each replica was set to 6 ns, leading to a total length of 192 ns simulation length for all effective temperatures.

A similar procedure was employed for the unbound state, where the unbound guest was sampled in vacuo. The relevant parameters were adjusted under this condition, e.g., the number of replicas (12) and the high‐temperature limit (1800 K).

A key difference between the bound and unbound simulations was the inclusion of a restraining potential in the bound‐state ensemble, which was added to improve the convergence of the absolute free energy calculation. Here, the restraining regime (imposed between centers of masses) employed in many references, e.g., by Piero Procacci and co‐workers were followed.^[^ ^19^ ^]^ Note that this retraining potential also remains in the lateral nonequilibrium Hamiltonian switching process and would be removed in post‐simulation processing.

Second, a swarm of nonequilibrium fast‐switching simulations was initiated from the equilibrium configurations extracted from the room‐temperature replica (i.e., the experimental condition). For both the bound and unbound states, 150 samples/trajectories were considered. The Hamiltonian switching process was decomposed into two steps, i.e., the charge and vdW switching sub‐steps. The bound‐state transition starts by gradually switching off the electrostatics of the guest molecule (i.e., discharging), after which the vdW component of the guest vanishes. As for the unbound‐state leg, the system starts with the guest‐solvent uncoupled condition, and the guest‐solvent interactions were gradually turned on (first vdW and then electrostatics). The Hamiltonian switching was performed at each time step, and the lengths of the bound‐state annihilation and the unbound‐state growth processes last 1.4 ns (0.4 ns electrostatics and 1 ns vdW) and 0.6 ns (0.4 ns vdW and 0.2 ns electrostatics). Such a length, according to our test, could generally lead to converged estimates through the ordinary free energy perturbation estimator (i.e., exponential averaging),

(6) $$\Delta G_{calc,\;step}=-\frac{1}{\beta }\ln{\langle e^{-\beta W}\rangle }_{step}$$

where the subscript step could be the bound‐state guest annihilation or the unbound‐state guest creation. The final estimate of Δ*G_calc_* can be obtained by combining the estimates of individual steps (Δ*G*_*calc*, *bound*_ *and* Δ*G*_*calc*, *unbound*_).

While asymptotically unbiased, the statistical behavior of unidirectional perturbation was still often less satisfactory. An additional numerical treatment called work convolution was therefore conducted,^[^ ^19^, ^20^, ^38^ ^]^ which combines independent microscopic works from the bound‐ and unbound‐state alchemical switching.

(7) $$\Delta G_{calc}=-\frac{1}{\beta }\ln\frac{1}{n_{bound}n_{unbound}}\sum_{m,n}^{n_{bound}*n_{unbound}}e^{-\beta W_{mn}}$$

where *n* denotes the number of works/realizations. Although the convolution sampling treatment would not increase the number of independent samples, it would smooth statistical fluctuations by averaging all cross‐combinations of microscopic works, leading to smoother and more stable exponential averages. Further, it improves numerical stability and convergence, especially for systems with broad energy distributions.

Third, the above‐obtained calculated binding strength requires corrections in order to compensate for various mismatches or differences between the standard‐state condition and the molecular simulation. The first correction term in Equation (4) was the volume mismatch, where the bound‐state volume *V_site_* should be adjusted to the standard‐state *V*_0_

(8) $$\Delta \;G_{volume}=-\frac{1}{\beta }\ln\frac{V_{0}}{V_{site}}$$

This concentration correction can be computed analytically through the restraining potential imposed during the bound‐state simulations. For this treatment, the restraining potentials between centers of mass employed in many references were followed.^[^ ^19^ ^]^

The charge correction Δ*G_charge_* accounts for the finite‐size artefacts in the discharging of charged molecules under periodic boundary conditions with lattice sums. The exact formula of the charge correction term depends on the neutralization treatment in the alchemical simulations.^[^ ^39^ ^]^ In our situation with a neutralized bound state and a non‐neutralized unbound state, the correction term could be written as

(9) $$\Delta \;G_{charge}=332*\pi *\left(-\frac{q_{guest}^{2}}{2\alpha^{2}V_{box,\;unbound}}-\frac{q_{guest}^{2}}{2\alpha^{2}V_{box,\;bound}}\right)$$

where $q_{guest}$ denotes the net charge of the drug‐like guest, *V_box_* was the size of the simulation box, the subscripts bound and unbound were self‐explanatory, and α represents the Ewald convergence parameter.

### Affinity Estimation Baselines: End‐Point Free Energy Calculations and Molecular Docking

Two types of commonly applied all‐atom modelling techniques were used for the estimation of the host‐guest/protein‐ligand binding strengths, the end‐point free energy calculation, and molecular docking.

Under the end‐point approximation, the binding affinity of the host and guest complex in solution can be expressed as

(10) $$\begin{array}{cc} \Delta G_{bind,\;solvated} & =\left(\Delta G_{host-guest,\;\;solv}-\Delta G_{host,\;solv}-\Delta G_{guest,\;solv}\right)\\ & \;+\Delta G_{bind,\;gas} \end{array}$$

where the subscripts were self‐explanatory. The solvation terms were estimated with the implicit‐solvent model, and the gas‐phase contribution was often computed with molecular mechanics (MM). Each solvation contribution involves two parts,

(11) $$\Delta \;G_{solv}=\Delta G_{solv,\;polar}+\Delta G_{solv,\;nonpolar}$$

The GB^[OBC 40,41]^ model was used for the polar part of the solvation contribution, while for the nonpolar part, the efficient linear solvent‐accessible surface area (SASA) regime was used^[^ ^40^, ^41^, ^42^ ^]^

(12) $$\Delta G_{solv,\;nonpolar}=\gamma *\Delta SASA$$

where the pre‐factor γ was a constant fitted with experimental values. For the gas‐phase contribution, the force‐field energetics, along with the normal mode analysis for the entropic estimate, were incorporated

(13) $$\Delta \;G_{bind,\;gas}=\Delta E_{elec}+\Delta E_{vdW}-T\Delta S_{gas}$$

This leads to the widely known and commonly applied MM/GBSA regime.

Aside from the ordinary or naïve MM/GBSA implementation, a more advanced regime called QM/GBSA was also considered, where the force‐field part was replaced by the multi‐scale QM treatment. Here, the whole solute system was included in the QM region, and three common semi‐empirical QM selections were selected, including AM1, PM6, and DFTB3. Consequently, the names of these multi‐scale QM/GBSA regimes were AM1/GBSA, PM6/GBSA, and DFTB3/GBSA. A final note to add was that the configurations used in the end‐point free energy calculations were the 150 configurations explored during the REST2 sampling of the bound state, in order to achieve an exact matching (in terms of configurations used in free energy extraction) with the nonequilibrium fast‐switching transformation.

The second category of baseline regimes was molecular docking. The well‐known open‐source AutoDock implementation called AutoDock Vina ^[^ ^29^ ^],^ using its default Vina and an altered Vinardo scoring functions ^[^ ^29^, ^30^, ^43^, ^44^ ^],^ and the smina^[^ ^45^ ^]^ implementation using its default scoring and fast scoring functions were considered. The cubic docking box was centered at the center of mass of the host cavity, and its side length was set to 12 Å.

### Interaction Analysis Based on GKS‐EDA(sol)

The GKS‐EDA(sol) method extends the original EDA‐PCM framework by incorporating the generalized Kohn–Sham (GKS) theory with an implicit solvation model.^[^ ^46^, ^47^ ^]^ In this approach, monomers were embedded into cavities within a dielectric medium, and the interaction between monomer charges and the solvent electric field was described by a reaction‐field operator, treated self‐consistently through the SCRF procedure. Within this framework, the total interaction free energy was decomposed into distinct contributions: electrostatic, exchange–repulsion, polarization, correlation, dispersion, and desolvation.

(14) $$\Delta G^{total}=\Delta G^{ele}+\Delta G^{exrep}+\Delta G^{pol}+\Delta G^{corr}+\Delta G^{disp}+\Delta G^{desol}$$

For a supramolecule *S* consisting of several monomers *M* (host and guest molecules), the individual terms were defined as

(15) $$\Delta G^{ele}=\langle \Phi_{DP}^{SOL}\left|{\hat{F}}_{S}^{D}\right|\Phi_{DP}^{SOL}\rangle \;-\sum_{M}\langle \Phi_{M}^{SOL}\left|{\hat{F}}_{M}^{D}\right|\Phi_{M}^{SOL}\rangle$$

(16) $$\Delta G^{exrep}=\langle \hat{A}\Phi_{DP}^{SOL}\left|{\hat{F}}_{S}^{D}\right|\hat{A}\Phi_{DP}^{SOL}\rangle \;-\langle \Phi_{DP}^{SOL}\left|{\hat{F}}_{S}^{D}\right|\Phi_{DP}^{SOL}\rangle$$

(17) $$\Delta G^{pol}=\langle \Phi_{S}^{SOL}\left|{\hat{F}}_{S}^{D}\right|\Phi_{S}^{SOL}\rangle -\langle \hat{A}\Phi_{DP}^{SOL}\left|{\hat{F}}_{S}^{D}\right|\hat{A}\Phi_{DP}^{SOL}\rangle$$

(18) $$\Delta G^{corr}=\langle \Phi_{S}^{SOL}\left|{\hat{\nu }}_{C}^{GKS}\left(S\right)\right|\Phi_{S}^{SOL}\rangle \;-\sum_{M}\langle \Phi_{M}^{SOL}\left|{\hat{\nu }}_{C}^{GKS}\left(M\right)\right|\Phi_{M}^{SOL}\rangle$$

(19) $$\Delta G^{desol}=\frac{1}{2}\;\langle \Phi_{S}^{SOL}\left|{\hat{\nu }}^{SOL}\right|\Phi_{S}^{SOL}\rangle -\frac{1}{2}\sum_{M}\langle \Phi_{M}^{SOL}\left|{\hat{\nu }}^{SOL}\right|\Phi_{M}^{SOL}\rangle$$

(20) $$\Delta G^{disp}=\Delta G_{S}^{disp}\;-\sum_{M}\Delta G_{M}^{disp}$$

Here, $\Phi_{S}^{SOL}$ and $\Phi_{M}^{SOL}$ represent the solute wavefunctions of the supramolecule and monomers optimized under the SCRF procedure within PCM. $\hat{A}$ denotes the antisymmetrization operator, while ${\hat{\nu }}^{SOL}$ corresponds to the PCM‐defined reaction field and cavity construction method. For the GKS‐EDA calculations, the XACS webserver was used.^[^ ^48^ ^]^

## Conflict of Interest

There are no conflicts of interest to declare.

## Author Contributions

X. W. did data curation, formal analysis, funding acquisition, investigation, methodology, resources, software, validation, visualization, and writing – review & editing. L. Q. did formal analysis, funding acquisition, investigation, resources, software, writing – review & editing. H. W. did formal analysis, investigation, resources, software, writing – review & editing. W. T. did formal analysis, investigation, resources, software, Writing – review & editing. J. L.did formal analysis, investigation, resources, software, Writing – review & editing. J. Z. H. Z. did data curation, formal analysis, funding acquisition, investigation, methodology, project administration, resources, software, Supervision, and writing – review & editing. P. P. did formal analysis, funding acquisition, investigation, methodology, project administration, resources, software, supervision, writing – review & editing. Z. S. did conceptualization, data curation, formal analysis, funding acquisition, investigation, methodology, project administration, Resources, software, supervision, validation, visualization, Writing – original draft, Writing – review & editing.

## Supporting information



Supporting Information

Supporting Information

Supporting Information

## Data Availability

All software employed in this work are freely accessible for academic use. We share the AMBER parameters of the refitted force field and the bound structures of all host‐guest pairs obtained under the molecule‐specific FM parameters in the supporting information.
