# Supplementary material for: Achieving Chemical Accuracy in Cyclodextrin Host–Guest Binding via Integrative Atomistic Modelling
Source: Adv Sci (Weinh). 2025 Dec 14;13(10):e19782. doi: 10.1002/advs.202519782 (PMC12915143; doi:10.1002/advs.202519782)
Supplement: Supplementary file 3 — Supporting Information [file ADVS-13-e19782-s002.pdf]

Supporting Information

Achieving Chemical Accuracy in Cyclodextrin Host–Guest Binding via Integrative Atomistic Modelling

Xiaohui Wang[1], Linqiong Qiu[2], Hongyu Wang[1], Wenting Tang[3], Jiayang Leng[4], John Z. H. Zhang[1,5,6*], Piero Procacci[7*], and Zhaoxi Sun[1*]

[1]*Faculty of Synthetic Biology, Shenzhen University of Advanced Technology, Shenzhen 518107, China*

[2]*Faculty of Biosciences, Taizhou Technician College, Zhejiang, 318000, China*

[3]*Beijing Key Laboratory of Digital Media, School of Computer Science and Engineering, Beihang University, Beijing 100191, China*

[4]*Department of Biomedical Engineering, Southern University of Science and Technology, Shenzhen, Guangdong, China*

[5]*Shanghai Frontiers Science Center of Artificial Intelligence and Deep Learning and NYU-ECNU Center for Computational Chemistry, NYU-Shanghai, 1555 Century Avenue, Pudong New Area, Shanghai 200062, China*

[6]*Department of Chemistry, New York University, NY, NY 10003, USA*

[7]*Dipartimento di Chimica "Ugo Schiff", Università degli Studi di Firenze, Via della Lastruccia 3, 50019 Sesto Fiorentino, Italy*

*To whom correspondence should be addressed:

John Z. H. Zhang john.zhang@nyu.edu

Piero Procacci piero.procacci@unifi.it

Zhaoxi Sun z.sun@suat-sz.edu.cn

S1. The host-guest dataset.

The experimental 1:1 binding constants used in this study were collected from established aggregated sources, including the CD dataset used to develop machine learning estimators, entries from the Suprabank database, SAMPL host-guest challenges, and additional values obtained via personal communication.[1-9] Because these datasets were originally compiled from multiple experimental studies, individual primary references are not available for all entries. To ensure consistency, we retained only guest molecules with room-temperature measurements (298-300 K) and more importantly with binding affinities reported for all of the three prototypical CD hosts (i.e., α-, β-, and γ-CD). Consequently, a total of 222 entries involving 74 unique guests binding to all of the three hosts are secured. Although individual bibliographic links cannot be fully reconstructed, all data originate from reputable curated sources, and the dataset-level provenance is documented for transparency.

Fig. S1. Guest molecules binding to cyclodextrins. The experimental binding strengths on α-CD is given under the molecular graphs, in kcal/mol.

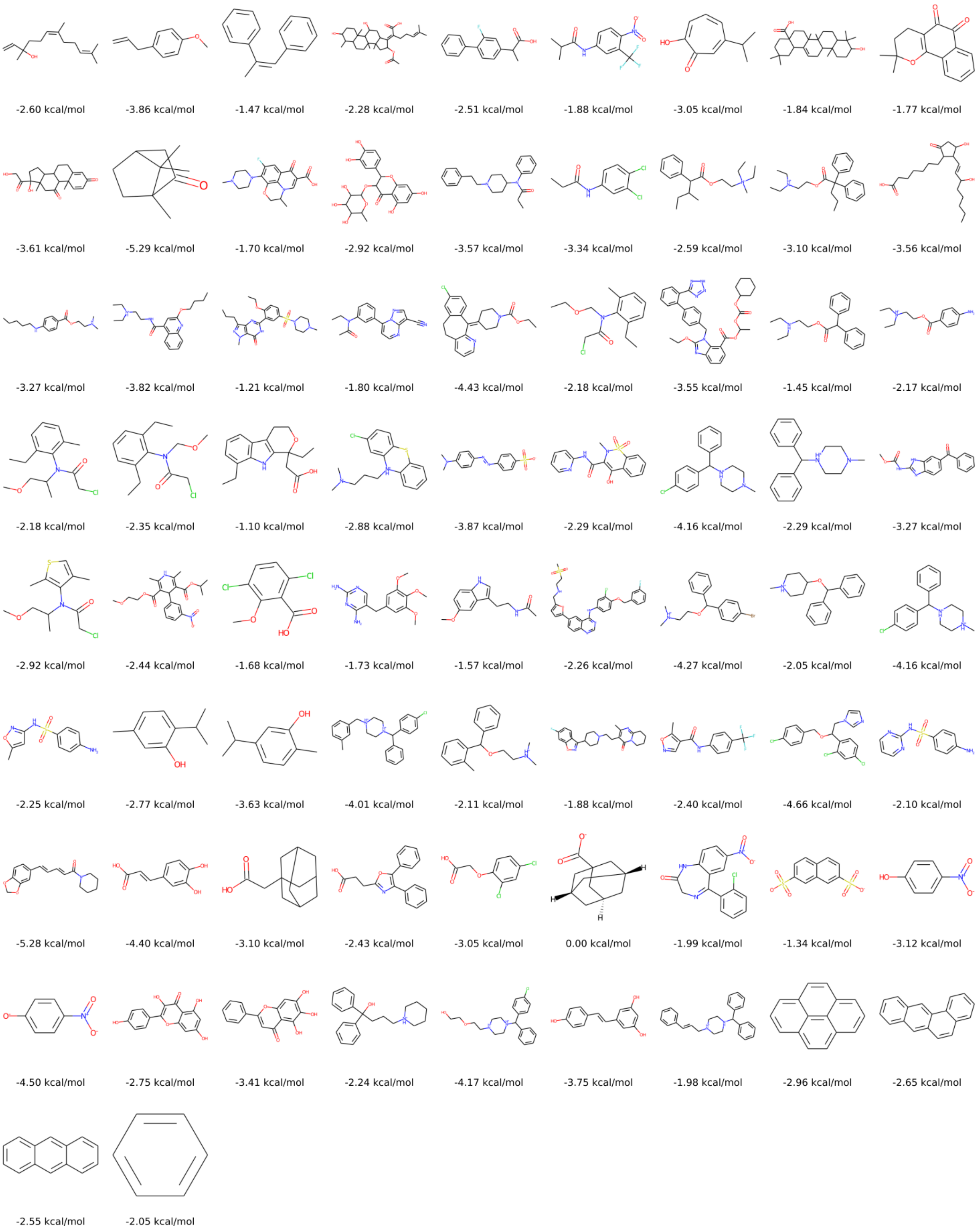

Fig. S2. Guest molecules binding to β-CD, with affinities shown in kcal/mol.

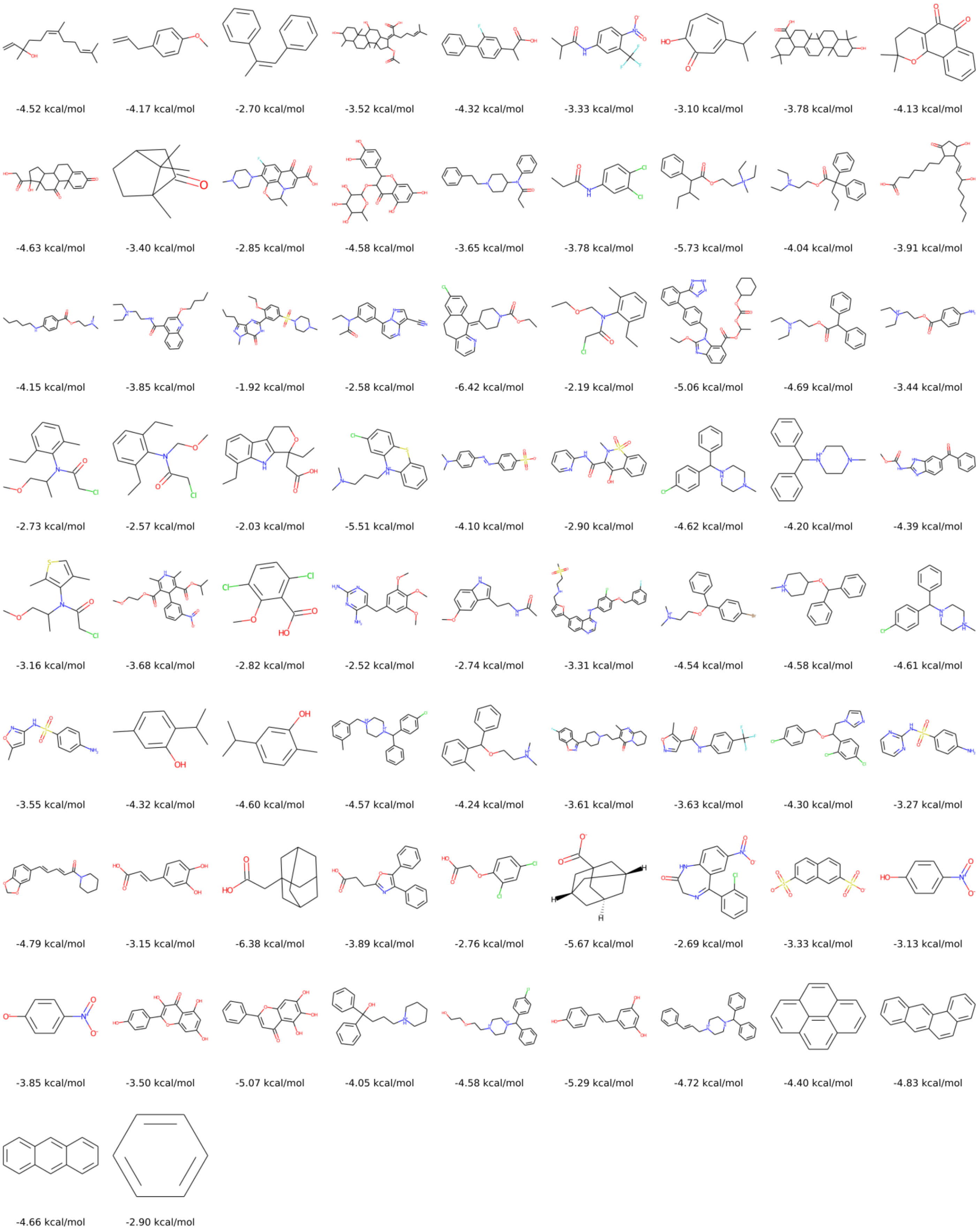

Fig. S3. Guest molecules binding to γ-CD, with affinities shown in kcal/mol.

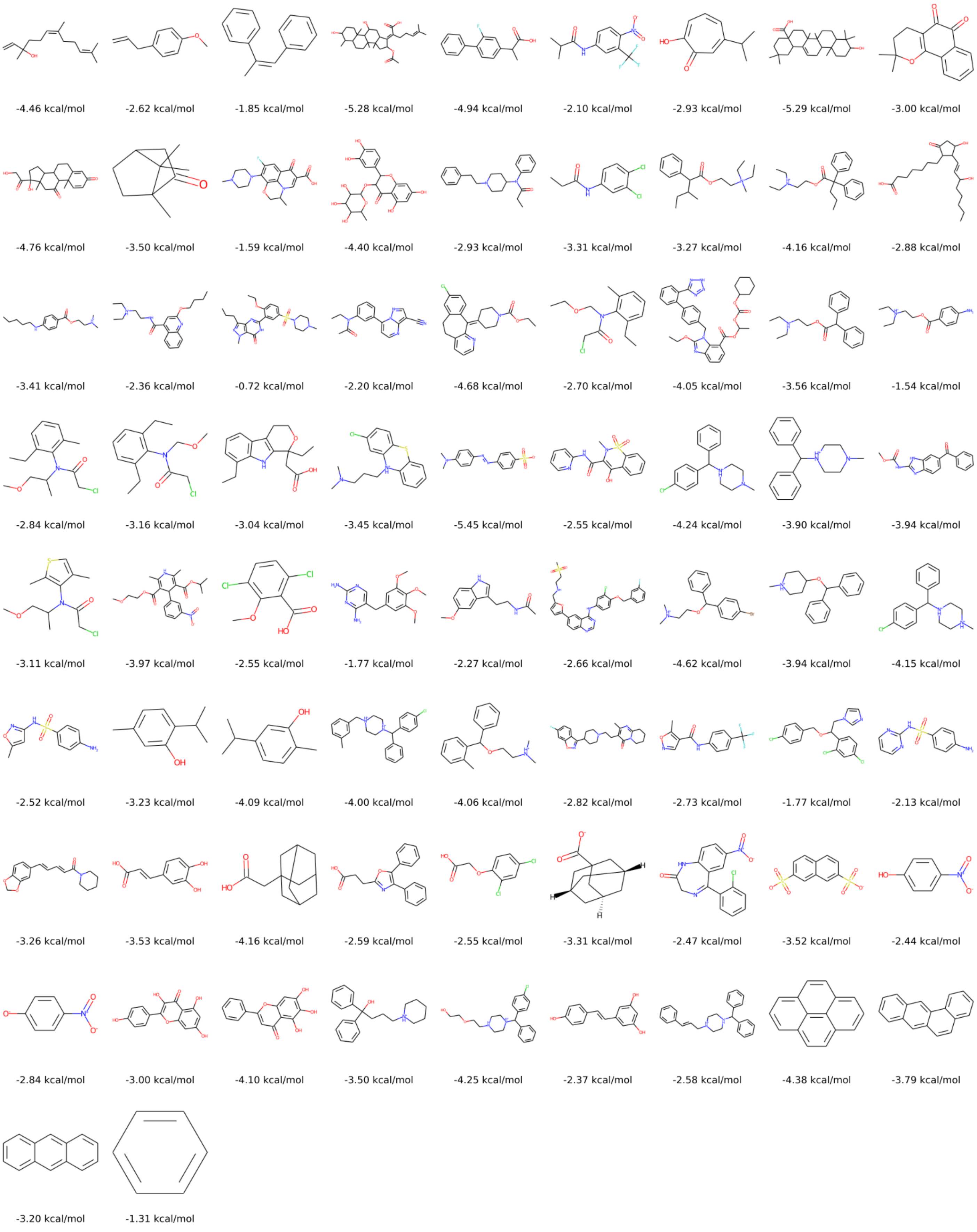

Fig. S4. Performance metrics for the α-CD host-guest dataset. MAE is in kcal/mol, while the other are dimensionless.

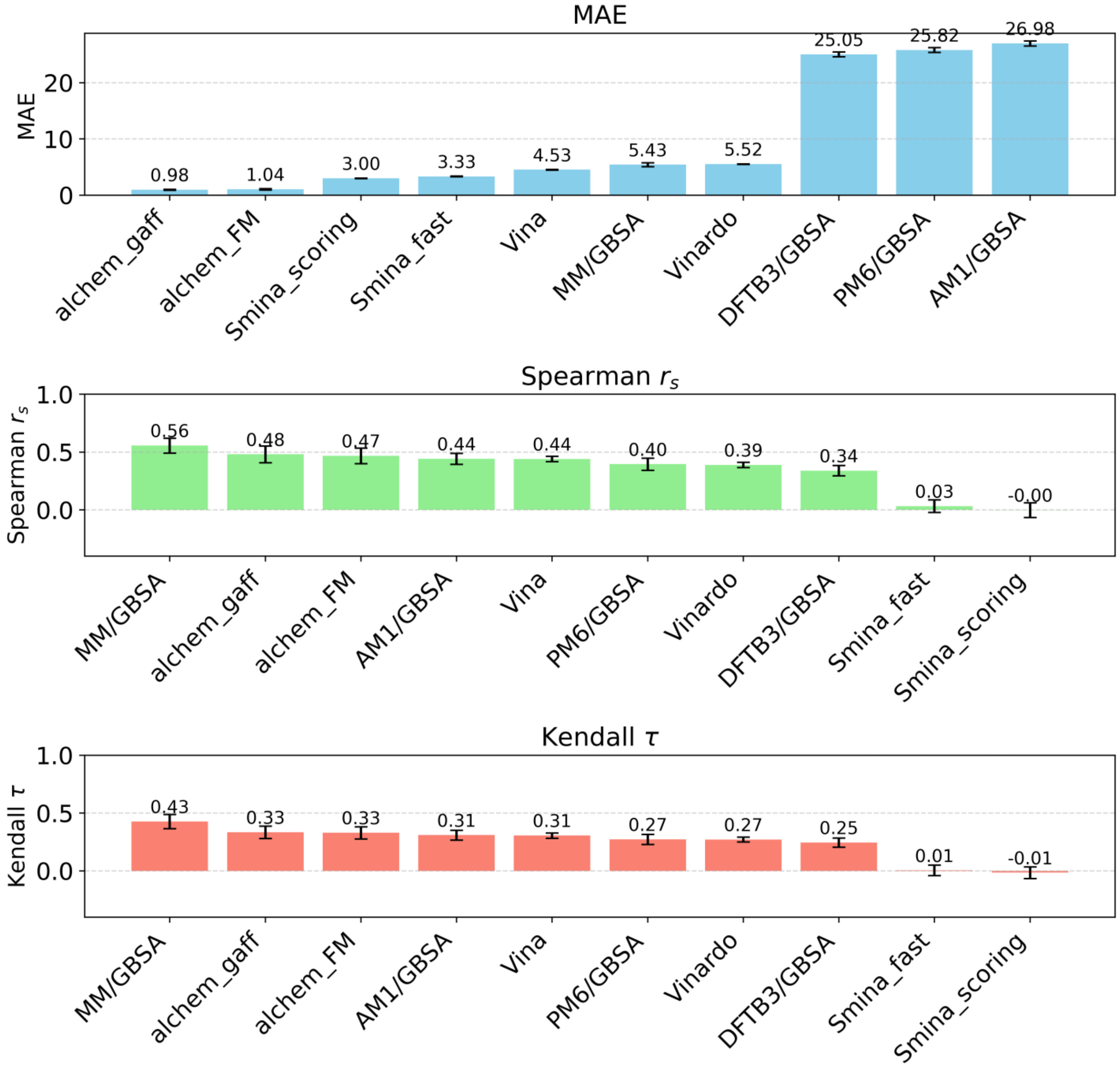

Fig. S5. Performance metrics for the β-CD host-guest dataset. MAE is in kcal/mol, while the other are dimensionless.

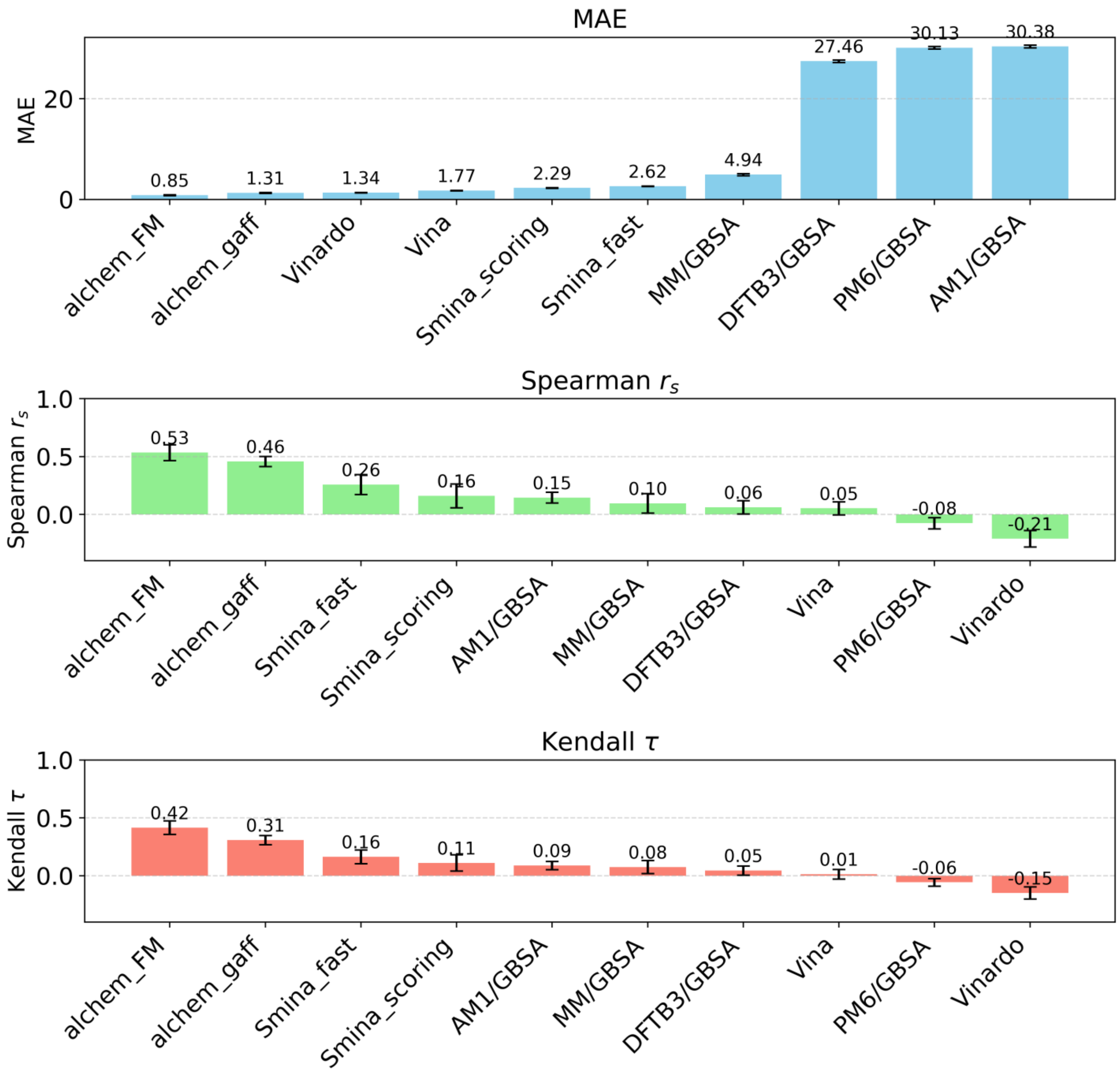

Fig. S6. Energy components from GKS-EDA analysis for the two binding poses shown in Fig. 3B.

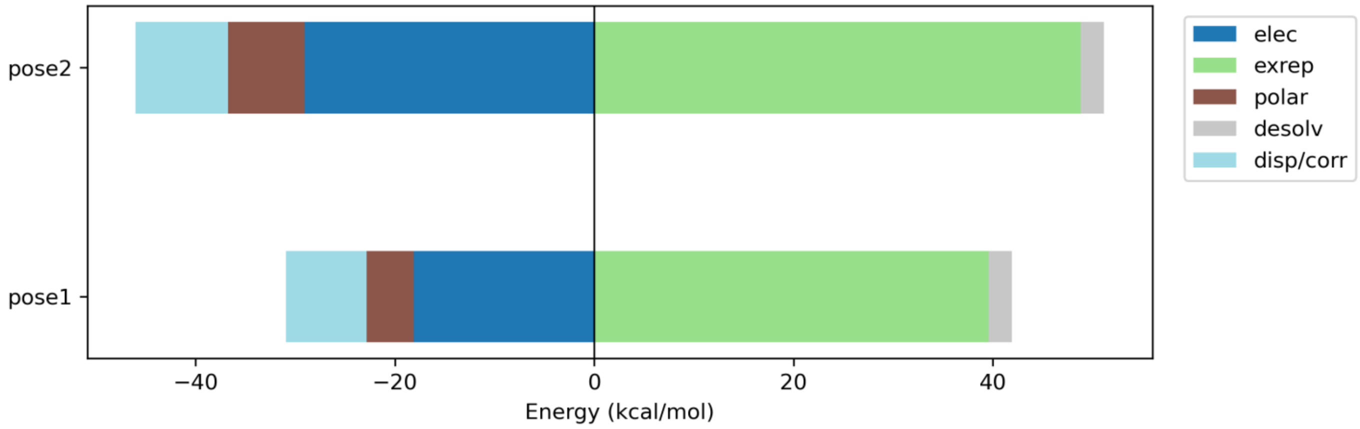

Tab. S1. Comparison between the computational cost of the new alchemical-based protocol and the sampling strategy simulating the binding/unbinding event along the physical pathway. The GPU hardware is RTX 4090 and the simulation engine is GROMACS 2020.6 patched with PLUMED 2.7.4.

simulation cells		number of atoms	sampling time (ns)	GPU hours
Alchemical	gas-phase	~30	192	negligible
	unbound	~4000	90	0.83077
	bound	~8000	402	6.432
Physical		~30000	1000	32

References

1. https://suprabank.org/.
2. Martin, A.; David, M., *SAMPL7 Challenge Overview: Assessing the Reliability of Polarizable and Non-Polarizable Methods for Host-Guest Binding Free Energy Calculations*. 2020.
3. https://github.com/samplchallenges/SAMPL9.
4. Kellett, K.; Slochower, D. R.; Schauperl, M.; Duggan, B. M.; Gilson, M. K., Experimental characterization of the association of β-cyclodextrin and eight novel cyclodextrin derivatives with two guest compounds. *J. Comput.-Aided Mol. Des.* **2020**.
5. Serillon, D.; Bo, C.; Barril, X., Testing automatic methods to predict free binding energy of host–guest complexes in SAMPL7 challenge. *J. Comput.-Aided Mol. Des.* **2021**, 35, 209-222.
6. Mizera, M.; Muratov, E. N.; Alves, V. M.; Tropsha, A.; Cielecka-Piontek, J., Computer-aided discovery of new solubility-enhancing drug delivery system. *Biomolecules* **2020**, 10, 913.
7. Jeschke, S.; Cole, I. S., 3D-QSAR for binding constants of β-cyclodextrin host-guest complexes by utilising spectrophores as molecular descriptors. *Chemosphere* **2019**, 225, 135-138.
8. Ahmadi, P.; Ghasemi, J. B., 3D-QSAR and docking studies of the stability constants of different guest molecules with beta-cyclodextrin. *J. Inclusion Phenom. Macrocyclic Chem.* **2014**, 79, 401-413.
9. Zhao, Q.; Ye, Z.; Su, Y.; Ouyang, D., Predicting complexation performance between cyclodextrins and guest molecules by integrated machine learning and molecular modeling techniques. *Acta Pharmaceutica Sinica B* **2019**, 9, 1241-1252.
